# Nanopore-Based Direct RNA-Sequencing Reveals a High-Resolution Transcriptional Landscape of Porcine Reproductive and Respiratory Syndrome Virus

**DOI:** 10.3390/v13122531

**Published:** 2021-12-16

**Authors:** Riteng Zhang, Peixin Wang, Xin Ma, Yifan Wu, Chen Luo, Li Qiu, Basit Zeshan, Zengqi Yang, Yefei Zhou, Xinglong Wang

**Affiliations:** 1 College of Veterinary Medicine, Northwest A&F University, Xianyang 712100, China; 2016107065@njau.edu.cn (R.Z.); 2514429383@nwafu.edu.cn (P.W.); max_zzzz@163.com (X.M.); 18392109960@163.com (Y.W.); 2020050551@nwafu.edu.cn (C.L.); QL525@126.com (L.Q.); yzq1106@nwsuaf.edu.cn (Z.Y.); 2 Department of Microbiology, Faculty of Life Sciences, University of Central Punjab, Johar Town, Lahore 54000, Pakistan; dr.basitzeshan@ucp.edu.pk; 3 Department of Life Science, Nanjing Xiaozhuang University, Nanjing 211171, China

**Keywords:** PRRSV, transcriptional regulatory sequences, transcriptomics, nanopore, epitranscriptome

## Abstract

The TRS-mediated discontinuous transcription process is a hallmark of Arteriviruses. Precise assessment of the intricate subgenomic RNA (sg mRNA) populations is required to understand the kinetics of viral transcription. It is difficult to reconstruct and comprehensively quantify splicing events using short-read sequencing, making the identification of transcription-regulatory sequences (TRS) particularly problematic. Here, we applied long-read direct RNA sequencing to characterize the recombined RNA molecules produced in porcine alveolar macrophages during early passage infection of porcine reproductive and respiratory syndrome virus (PRRSV). Based on sequencing two PRRSV isolates, namely XM-2020 and GD, we revealed a high-resolution and diverse transcriptional landscape in PRRSV. The data revealed intriguing differences in subgenomic recombination types between the two PRRSVs while also demonstrating TRS-independent heterogeneous subpopulation not previously observed in Arteriviruses. We find that TRS usage is a regulated process and share the common preferred TRS in both strains. This study also identified a substantial number of TRS-mediated transcript variants, including alternative-sg mRNAs encoding the same annotated ORF, as well as putative sg mRNAs encoded nested internal ORFs, implying that the genetic information encoded in PRRSV may be more intensively expressed. Epigenetic modifications have emerged as an essential regulatory layer in gene expression. Here, we gained a deeper understanding of m5C modification in poly(A) RNA, elucidating a potential link between methylation and transcriptional regulation. Collectively, our findings provided meaningful insights for redefining the transcriptome complexity of PRRSV. This will assist in filling the research gaps and developing strategies for better control of the PRRS.

## 1. Introduction

Porcine reproductive and respiratory syndrome (PRRS) has been one of the most economically significant infectious diseases that seriously affected the global swine industry over the past three decades [1]. The etiologic agent, PRRS virus (PRRSV), is an enveloped single-stranded positive-sense RNA virus classified within the order *Nidovirales*, family *Arteriviridae*. The current family *Arteriviridae* is further specified into six subfamilies, including Variarterivirinae, Zealarterivirinae, Equarterivirinae, Simarterivirinae, Crocarterivirinae, and Heroarterivirinae [2,3]. The viral genome of PRRSV is approximately 15 kb in length and comprises at least 10 open reading frames (ORFs). The genomic RNA features two large overlapping open reading frames (ORF1a and ORF1b) in the 5′ end that auto-proteolytically cleaved to yield 16 non-structural proteins (nsps) later, the majority of which act as a driving force for PRRSV genome replication and subgenomic mRNA transcription [2,4]. The 3′ end of the genome contains ORFs 2-7 that are translated from the nested sets of 3′ and 5′ co-terminal subgenomic mRNAs (sg mRNAs) [5,6,7].

By infecting the monocyte-macrophage lineage, especially preferentially in pulmonary alveolar macrophages (PAMs), the conditions may develop into severe proliferative and interstitial pneumonia. The International Committee on Taxonomy of viruses grouped into two genotypes: European (Type 1, Lelystad) and North American (Type 2, VR-2332). Nowadays, multiple genetic lineages coexist in Chinese swine herds, including lineage 1 (NADC30-like), 3 (QYYZ-like), 5 (VR2332-like), and 8 ((CH-1a-Like/JXA1-like) of type 2 PRRSV [8,9]. As RNA-dependent RNA polymerase (RdRp) lacks the 3′ to 5′ proofreading exonuclease activity, considerable genetic heterogeneity and taxonomic diversity have been noted among the PRRSV strains fully sequenced to date. The mutational spectrum of genotypes rather than individual variants was thought to be the underlying molecular mechanisms of evolutionary events (termed quasispecies) [10,11].

As proposed by Sawicki and van Marle, the transcription hallmark of PRRSV was a discontinuous elongation of minus strands to eventually produce a series of sg mRNAs via template switch events [7,12]. During negative-strand RNA synthesis, the viral replication and transcription complex (RTC) interrupts transcription following the encounter of body TRS (TRS-B) and is re-initiated at the leader sequence (TRS-L) located at the 5′ end. The consensus TRS-L core motif of PRRSV is 5′-UUAACC-3′, and six major sg mRNAs (sg mRNA 2-7) have been reported to be produced in PRRSV-infected cells; for more details, please refer to [13,14,15]. However, despite these sg mRNAs being thought to be an indicator of effective viral infection and flexible transcriptional activity, their abundance and complexity in host cells have been grossly overlooked [16]. 

Among different molecular platforms, conventional next-generation sequencing (NGS) has played an essential role in elucidating the transcriptomics of SHFV-infected cells [17,18]. Nevertheless, this approach has its limitations, such as the inability to accurately characterize full-length transcriptomes with intact 3′ and 5′ termini, making it difficult to define the configuration of sg mRNAs or the specific TRS type in recombination events. Indeed, Nanopore sequencing technology (ONT) is capable of generating ultra-long reads and has been developed for the characterization of the highly nested transcriptomic complexity of various viruses [19,20]. In this work, we applied for the first time the Nanopore platform to provide a deep characterization of splicing events generated in PRRSV at a high-resolution transcriptome-wide, and these molecules presented an extensive and similar recombination pattern during replication in vitro. Our analyses emphasized the differential transcriptional activity in porcine alveolar macrophages with two distinct strains and further offered more accurate insights into global body TRS sites. The discovery of multiple novel splice isoforms means that the coding capacity of PRRSV was more complex than previously anticipated. Therefore, further experiments of the targets are still needed to validate them in the future.

The data also offered critical insight into epigenetic modification at the transcriptomic level. Both viruses induced different epigenetic responses in infected PAMs. In addition, a consistent pattern of methylation modification of different sg mRNAs relative to the genome was observed. In general, these findings provide a basis for future research of its function and biological significance in the transcription process.

## 2. Materials and Methods

### 2.1. Sample Preparation and Total RNA Extraction

The 6-week-old specific pathogen-free (SPF) piglet was euthanized, and the porcine alveolar macrophages (PAMs) were obtained from lung lavage, as previously described [21]. The two different PRRSV isolates sequenced in this study were XM-2020 (MZ160905.1, lineages 1) and GD (EU109503.1, lineages 8), respectively. PAMs were inoculated with PRRSV at a multiplicity of infection (MOI) of 1 in serum-free media (RPMI 1640, Gibco BRL Co., Ltd., Grand Island, NY, USA) and incubated for 12 h at 37 °C. After media removal, cell pellets were immediately frozen in liquid nitrogen for 2 h, then stored at −80 °C for RNA extraction (R6834 Total RNA Kit I, Omega Bio-Tek, Inc., Norcross, GA, USA). The RNA concentration and purity of each sample were checked using the NanoDrop One spectrophotometer (Thermo Scientific, Wilmington, DE, USA) and a Qubit 2.0 Fluorometer (Invitrogen, Carlsbad, CA, USA).

To determine the initial replication of PRRSV in host cells, the viral titers were calculated at 12 h post-inoculation (hpi) by the method of Reed-Muench. Six independent biological and three technical replicates were performed for each sample. Differences between groups were assessed by one-way ANOVA (*p* values below 0.05 were considered significant).

### 2.2. Library Preparation and Sequencing

Nanopore direct RNA sequencing: Direct RNA libraries were prepared according to the manufacturer’s instructions (SQK-RNA002, Oxford Nanopore Technologies, Oxford, UK) and loaded onto R9.4.1 SpotON flow cells, then sequenced on the PromethION device for 72 h [22]. Base-calling of raw data (fast5 format) was done by Guppy (v3.2.6) with default settings [23].

Illumina RNA sequencing: Following the manufacturer’s protocol (NEBNext^®^ UltraTM II kit, NEB, Ipswich, MA, USA), the Illumina workflow involved a tedious library construction process, including end repair and A-tailing, and adapter ligation and amplification. The purity and integrity of RNA libraries were evaluated with the Agilent 2100 Bioanalyser (Agilent Technologies, Inc., Santa Clara, CA, USA). Prepared denatured libraries were loaded onto the flow cell and then sequenced on a NovaSeq 6000 instrument (Illumina, San Diego, CA, USA). The read length of the paired-end approach to 2 × 150 bp.

### 2.3. Genome-Wide PRRSV Phylogenetic Analysis

Hybrid assembly was employed using both Nanopore and Illumina data by the MaSuRCA v3.2.9 hybrid assembler [24], and the Racon module was used to perform three rounds of error correction on the Nanopore sequencing data [25]. The assembled viral genome of 15 kb size was used as a framework for further analysis. To better understand the species distribution of the selected isolates, a phylogenetic tree was constructed based on the ClustalW method using the Molecular Evolutionary Genetics Analysis (Mega) version X program (The Biodesign Institute, Tempe, AZ, USA). All the PRRSV reference genomes were retrieved from GenBank, and the details are listed in Table S1.

### 2.4. Data Analysis

Nanopore: NanoFilt v.2.7.1 was used to further trim and filter out low-quality sequencing reads [26]. Then, FMLRC (v0.1.2) was used to perform hybrid correction on the filtered data, with the short-reads as a reference [27]. Raw reads were aligned to the reference genome of PRRSV by Minimap2 v2.11 (parameter: -ax splice -uf -k14 –secondary = no) [28]. Poor-quality and non-viral reads were filtered. Split-reads were selected using the CIGAR codes through an AWK command, and the coordinates of the breakpoint were recorded for further analysis. Samtools was used to identify intact sub-genomic mRNAs, and its read must map within the leader sequence of 30 bases (5′-GGTCTCTCCACCCCTTTAACCATGTCTGGG-3′). Body-TRS was identified by the hexamer 5′-UUAACC-3′ homology motif that occurred downstream of the splicing sites. 

Illumina: HISAT2 (v2.2.1) was used to align RNA-Seq data to the PRRSV reference strain and then identified the split reads by parsing the BAM alignment CIGAR. Partial default parameters were used in the analysis: --very-sensitive, --no-mixed, --no-discordant. Then, 5′ and 3′ selections for each spanning-junction read were recorded and then outputted all the contents as a tab-delimited.txt file for further analysis. All analyses were conducted using R software (version 4.0.3), and figures were plotted in ggplot2. 

### 2.5. Identification of 5mC Methylation

The threshold was set by the Q-score to execute the filtering command, and then the ont_fast5_api (parameter: -recursive version: 3.1.6) was used to convert the multi_read_fast5 file into the single_read_fast5 file. The 5mC methylation detection was configured using the following steps or parameters: Tombo v1.5 was first used to resquiggle raw nanopore reads (parameter: default), and then ran using Tombo detect_modifications alternative_model command (parameter: --coverage-dampen-counts 2 0). We obtained the modification coverage per base in the final and used R for data analysis and visualization. See [26,29] for more details about Tombo commands and algorithms.

## 3. Results

### 3.1. The Prevalent Status and Genetic Diversity of PRRSV-2 in China

**Figure 1:**
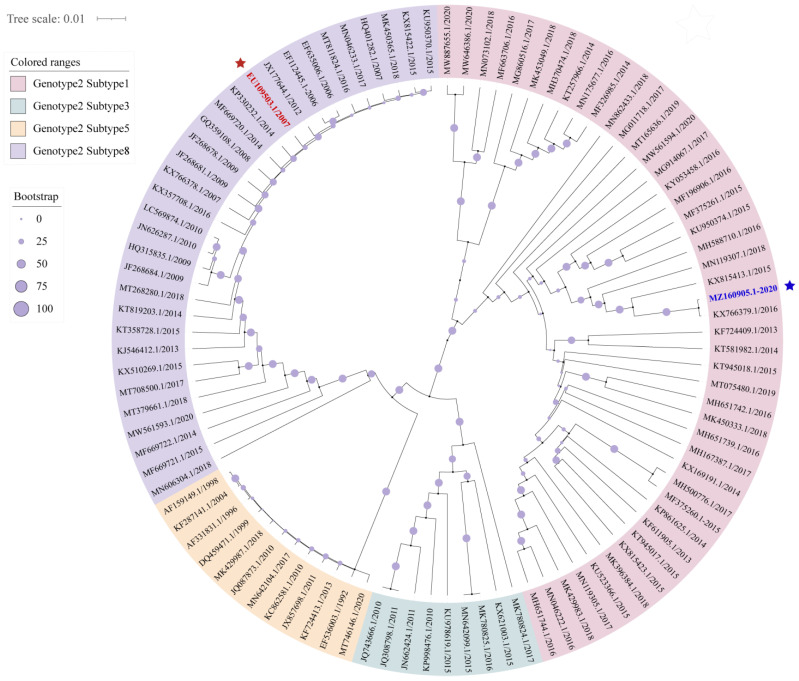
Whole genome-based phylogenetic analysis of type 2 PRRSV. Reconstructing the phylogenetic tree of the Neighbor-joining algorithm based on two clinical isolates and 96 reference strains (n = 98). Representative lineages marked with the corresponding color and bootstrap values were shown, including lineages 1 (pink), lineages 3 (green), lineages 5 (yellow), and purple (lineages 8). The collection_date of strain is shown behind the GenBank accession number, as indicated in the legend. Chinese XM-2020 and GD PRRSV isolates are labeled with blue and red, respectively.

Phylogenetic trees were constructed using 96 reference strains to explore the genetic diversity among PRRSV isolates. The prevalent PRRSV-2 strains showed a high degree of variability and were further clustered into four distinct lineages: lineage 1 (NADC30-like), lineage 3 (QYYZ-like), lineage 5 (VR2332-like), and lineage 8 (JXA1-like/HP-PRRSV). The phylogenetic investigations demonstrated that the two strains, XM-2020 (GenBank: MZ160905.1) and GD (GenBank: EU109503.1) used in the current study were designated as lineage 1 and lineage 8, respectively (Figure 1). Retrospective studies showed that the epidemic of lineage 8 reached its peak in 2009, after which it declined over time and was concomitant with a rapid increase in the occurrence of lineage 1 [11]. Compared to previously circulating PRRSV strains, the emerging variants were thought to possess higher inter-lineage recombination frequencies, thereby affecting the viral virulence and clinical outcome. It was still a puzzle how lineage 1 (NADC30-like) gradually replaced lineage 8 (HP-PRRSV) to become predominant. Additionally, this also posed a challenge for a high-throughput transcriptomics understanding of different evolutionary driving forces that can affect PRRSV genetic diversity. 

### 3.2. NADC30-Like PRRSV and HP-PRRSV Induce Different Transcriptional Activity during Infection in Susceptible PAM Cells

**Figure 2:**
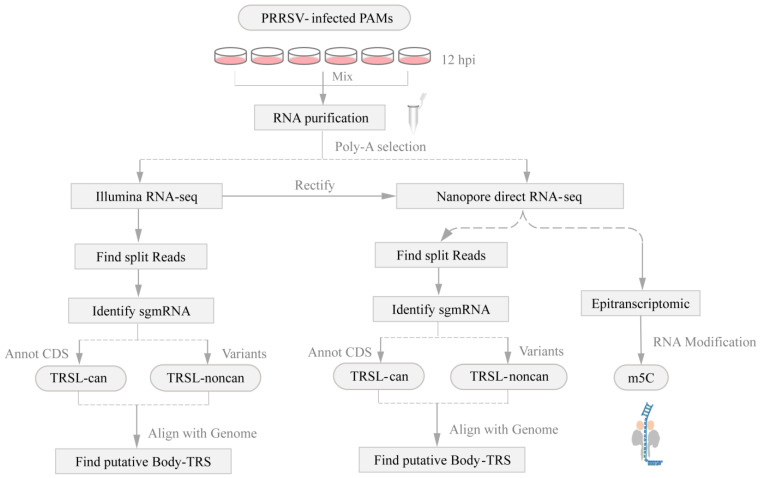
Schematic of the experimental workflow. The main pipeline is shown with gray text boxes and arrows. First, reconstructed subgenomic mRNA aligned with leader sequences based on analysis of different categories of split-reads. Second, systematically identified putative TRS-B sites in PRRSV genomes. Third, captured single-molecule resolution atlas of methylation modifications in native RNA molecules.

**Figure 3:**
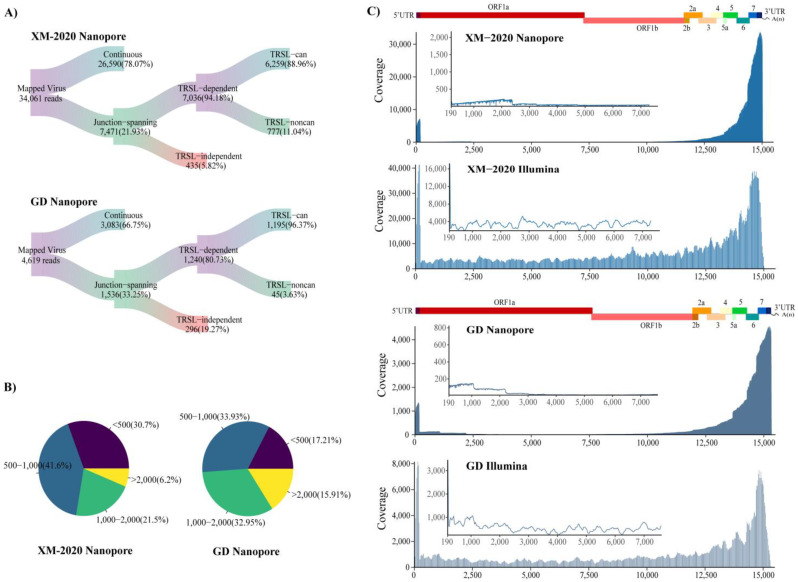
Sequencing data generated from two independent PRRSV isolates. (**A**) Various RNA molecules generated by native nanopore sequencing were evaluated and counted. Sankey diagram showed the read count of different splice isoforms categories. ‘Continuous’ represents the reads contiguously mapped to the viral reference genome. ‘Junction-spanning’ denotes the reads mapped to two variable splice sites up/downstream of the genome. Thereinto, the subset of reads that span over 30 nt leader sequence are designated as ‘TRSL-can’ while ‘TRSL-noncan’ represents the other fusions lacking the leader sequence. (**B**) Read length distributions. (**C**) Genome-wide coverage of distinct PRRSV isolates sequenced by Nanopore and Illumina technologies. The internal image magnifies the coverage for the non-structural gene.

To decode the transcriptomic profile of PRRSV during in vitro infection, the total libraries derived from 12 h of PRRSV-infected (or non-infected) porcine alveolar macrophages were sequenced on MinlON nanopore and Illumina Nova-seq, following the Workflow (Figure 2). Extracellular PRRSV RNA levels are shown in Figure S1. Mapping reads to these reference genomes yielded a total of 34,061 and 4619 unique transcripts in XM-2020 and GD samples, respectively, of which 7036 and 1240 chimeric RNAs contained leader sequence, respectively (Figure 3A; Table S2). The most abundant transcripts in both samples were centered between 500 and 1000 nt in length, particularly the longest transcript with a length of more than 10,000 nt, providing more precise insights into potential multi-mapping (Figure 3B). The analysis showed an intriguing difference between gRNA and sg mRNA reads in the depth of coverage, especially leading to a sharp drop after the leader sequence of the 5′UTR and followed by a gradual rise at the regions of structural proteins (Figure 3C). As expected, a high degree of the transcriptome complexity of PRRSV was attributed to multiple types of RNA splicing events. The majority of spliceosomes are the products of discontinuous transcription, which occurs between the leader sequence of the upstream 5′ UTR ends and the genome body (TRS-B). The canonical splicing donor is highly enriched in the annotated 5′ UTR ends. Beyond the 5′ and 3′ co-terminal canonical transcripts, some non-canonical products were detected, including non5_3, 5_non3, and non5_non3 (Figure S2). The latter suggests that the alternative splicing events are not simply controlled by the typical machinery of TRS-dependent (UUAACC motif), which means that potentially non-canonical transcriptional processes occurred during PRRSV infection. In addition, comparative transcriptome analysis revealed that the viral replication and transcriptional activity differ significantly among distinct strains. The transcriptional activity driven by XM-2020 infection seemed to be higher than compared to GD, and this heterogeneity may be caused by the differential ability in the early stages of virus replication. Detailed information about the chimeric RNAs identified by Nanopore and Illumina is supplied in Tables S2–S4. 

### 3.3. Analysis of Alternative Splicing Events during Transcription

**Figure 4:**
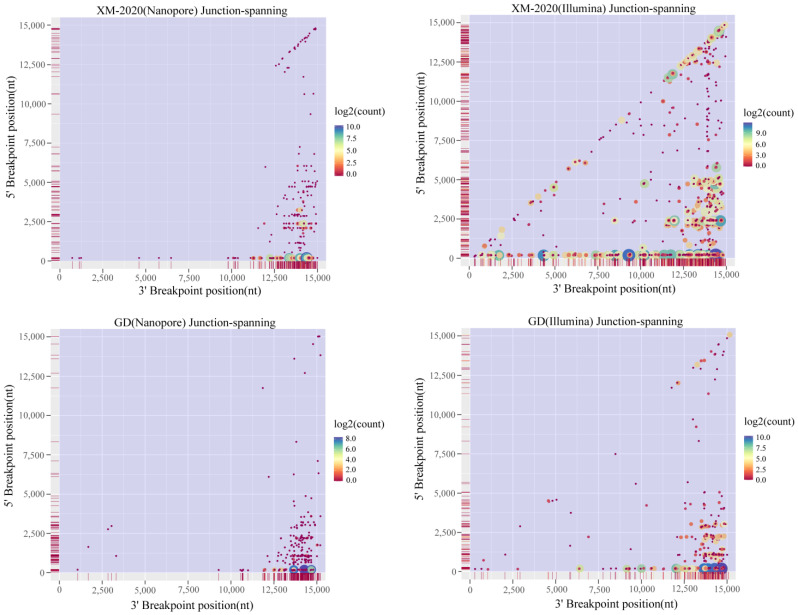
Multi-strategic sequencing for high-resolution RNA recombination map. The color indicates the number of mapped reads, with the axis scaled to log2. The start breakpoint on the X-axis corresponds to the end breakpoint of the Y-axis. The recombination frequency is marked with vertical red lines.

**Figure 5:**
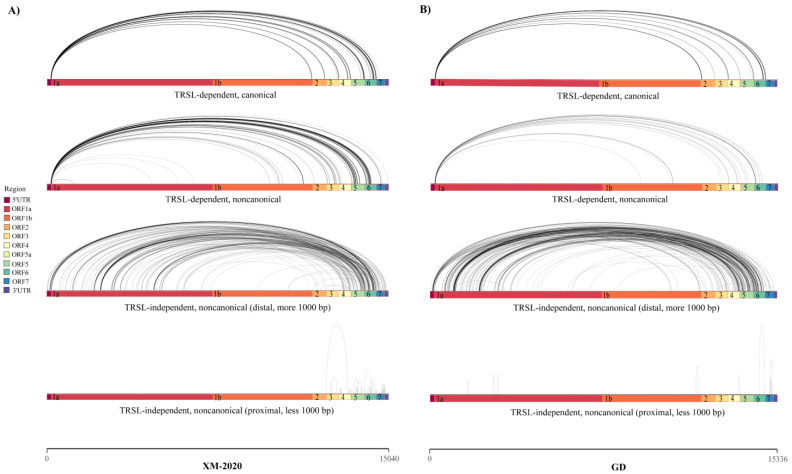
Global view of the RNA fusion events, which fall into four categories: (1) TRSL-canonical events. (2) TRSL non-canonical events. (3) TRSL-independent events with distal junctions. (4) TRSL-independent events with proximal junctions. These colored boxes represent the open reading frames. The split reads are displayed as black arcs over the viral genome sequence, and figures were created using the ggplot2 package in R ((**A**) in XM-2020, and (**B**) in GD).

Splicing events of the PRRSV RNAs, including the canonical (known) and non-canonical (novel isoforms), were further analyzed based on the collected data from either long-read Nanopore or short-read Illumina sequencing. The majority of splicing events identified by Nanopore could be readily recovered from Illumina data alone and were strongly supported by polyA data (Figure 4). Although the Illumina analysis identified larger numbers of isoforms, it was yet insufficient to cover the full scope of the sg mRNA diversity in length. As a consequence, the incomplete fragments of short read do not reflect the real abundance of the spliceosome in samples. The vast majority of the spliceosomes were generated by 5′ leader-dependent template switching between TRS-L and TRS-B (Figure 5A,B). The split reads mapped to the TRS-B encoding ORF7 protein were the most abundant of the TRSL-dependent canonical transcripts. Pronounced heterogeneity in the leader-body fusion sequence of the corresponding sg mRNAs was also observed. Further examination also revealed the existence of three types of discontinuous transcription events classified as the fusions of TRS-L to unexpected 3′ splicing sites, TRS-L independent long-distance fusions (gap more than 1000 nt), and TRS-L independent local fusions (gap less than 1000 nt) (Figure 5A,B).

**Figure 6:**
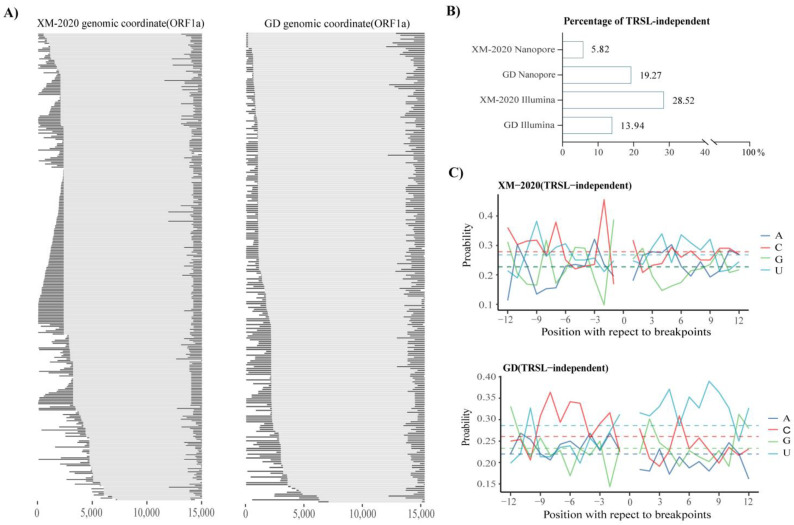
Characterization of TRSL-independent non-canonical events. (**A**) The fusion transcripts mapped to the ORF1a region were visualized. Black bars represent sequences that matched the viral genome, whereas dashed lines indicate the large deletions. (**B**) Proportions of the TRSL-independent events were categorized according to annotations of the 5′ junction sites, in four independent datasets. (**C**) The distribution of nucleotides in 12 nt regions upstream and downstream of the breakpoint in non-canonical splicing events. Abbreviations used: A = adenine, C = cytosine, G = guanine, U = uracil.

Intriguingly, these chimeric transcripts independent of TRS-L have not been appreciated as a distinct population before, and with differential expression between XM-2020 and GD (Figure 6A,B). The 5′ splicing sites of these heterogeneous subpopulations were more enriched upstream of the ORF1b without the hotspots pattern, while the 3′ junction sites were formed preferentially around transcriptionally active TRS-B loci. To assess whether such events are potentially related to some nucleotide preference surrounding breakpoint sites, sequence logos containing 12 upstream of the 3′ breakpoint and 12 downstream of the 5′ breakpoint nucleotides were generated. A strong preference for nucleotides surrounding the breakpoint sites was observed (Figure 6C). Cytosine (C) was favored upstream of the breakpoint, whereas the uracils (U) were favored downstream, suggesting that such features could be involved in the mechanism of non-canonical sg mRNAs generation. Although the potential mechanisms of aberrant RNA–RNA interactions are currently unknown and functionally might not code any viral proteins, they bring the viral transcriptome complexity to a higher level, which is worth more thorough studies to elucidate the machinery. 

### 3.4. Identification of TRS-B Sites in PRRSV Genome

**Figure 7:**
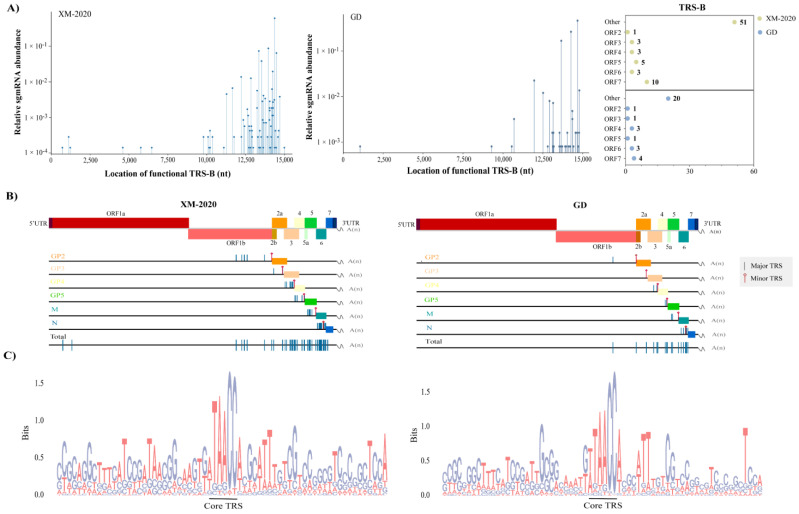
Genome-wide distribution of putative TRS-B sites. (**A**) The multi-phasic abundance of sub-genomic mRNA generated from individual TRS-B was shown. Peak height was positively correlated with the fractional usage of individual TRS-B. The cumulative count of TRS-B based on gene expression profiles is shown in the right part of the drawing. (**B**) A genome view of the putative TRS-B sites shared by Illumina and Nanopore methods was drawn. TRS with the highest fractional usage is marked as red bars. (**C**) The frequency of nucleotides surrounding all TRS-B sites was visualized using the SeqLogo package in R. TRS-B core sequences are noted with a black subscript.

All mapped TRS-dependent transcripts were analyzed for 3′ splice sequences by the nanopore data to identify putative TRS-B loci, most of which were located upstream in annotated ORFs. The utilization of individual TRS-B was calculated by dividing the relative abundance of the sg mRNAs it produced by the total number of transcripts from all TRSL-dependent transcripts. The highest density of TRS-B mapped to the 3′ structural protein region of the genome, of which the nucleocapsid protein (N) regulated by TRS7 was most abundant, generating 3324 reads in XM-2020 (~47.24%) and 579 reads in GD (~46.69%), respectively (Figure 7A). In combination with Nanopore and Illumina, 48 and 18 high-confidence shared loci of TRS-B were identified in XM-2020 and GD, respectively (Figure 7B). The distribution of TRS-B seems more complex than previously appreciated. The position deviation of TRS-B within the genome observed for different strains provided insights that each virus used specific molecular mechanisms during transcription. The body sequence composition showed that the nucleotide at position 1 had a higher frequency of single nucleotide (U-to-A) polymorphism in GD and XM-2020, while the nucleotides at positions 2–6 were relatively more conserved (Figure 7C). The above observations support our hypothesis that there is only one preferred TRS in each structural gene and that they are used normatively in transcriptional regulation. The newly identified minor TRS-B served as backup sites, generating additional sg mRNAs encoding the known structural proteins, but their abundance of transcripts was much lower than that from the major. By contrast, more backup TRS-B sites were identified in XM-2020. This low-level use of alternative TRS-B potentially enabled the virus to maximize the efficiency in terms of the optimal ratio of proteins required for assembly. Accordingly, the newly emerged lineage 1 was thought to drive PRRSV evolution through alternative TRS-B generation.

Multiple putative TRS-B were mapped to the non-structural protein region, where 14 and 4 sites were identified in XM-2020 and GD, respectively. These long sg mRNAs were expressed in ‘low’ abundance, which encoded different lengths of N-terminal-truncated polyproteins pp1a and pp1ab; most of these occurred within the ORF1b region rather than ORF1a. These distinctive patterns of transcription were shown in both viruses, indicating that subgenomic RNA synthesis is not restricted to the 3′-proximal areas of the genome throughout the transcriptional cycle. Consequently, the additional TRS-B within non-structural protein regions represented a source of reserve backups for those members of replication-transcription complex proteins lacking high transcription activity, especially nsp9, nsp10, and nsp11. These findings point to a potential selective pressure on TRS-B variants adapted to virus assembly.

Whether certain cis-acting elements in the viral genome can drive or facilitate the production of sg mRNA remains unclear. The sequence logo depicted the flanking residue distributions surrounding the identified TRS-B loci and found local position-specific motif preferences, e.g., a preference for ‘AUU’ residues was seen immediately 5–7 nt downstream of the body core sequence. These conserved flanking residues may function as cis-acting RNA elements to facilitate base pairing and template switching during sg mRNA synthesis (Figure 7C). 

The functional importance of the secondary structures of the body TRS regions has been reported in transcriptional responses of PRRSV [30,31]. It was found that the high-order structures of the TRS-B were similar to the 5′-proximal region and were all characterized by one putative stem-loop with the core sequence located at the top (Figure S3A). Conceivably, the template-switching between the 5′ leader and TRS-B may be a more parsimonious model to explain the formation of nascent negative-strand RNA during PRRSV transcription (Figure S3B). Therefore, its binding stability is required for efficient transcription. Consistent with this cognition, the nascent negative chain cTRS7 has a remarkably lower Gibbs binding free energy against the leader sequence, resulting in the highest stability of duplexes binding (Figure S3C). Additionally, sequence complementarity also existed between variable 5’ and 3’ flanking sequences and the leader sequence. All of the above characteristics will be conducive to enhancing the template-switching efficiency of nascent RNA chains, thus flexible hybridization with the leader TRS during discontinuous transcription.

### 3.5. Gene Predictions and Annotations

**Figure 8:**
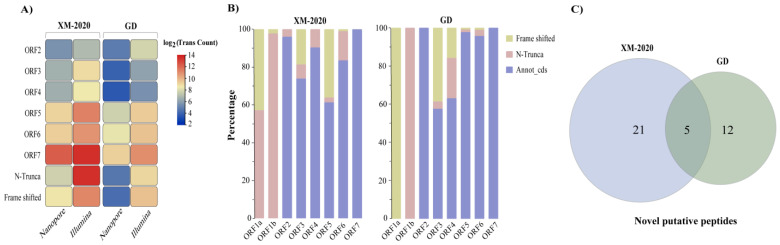
Coding gene prediction and functional annotation within PRRSVs. The NBCI ORF finder tool was used to search open reading frames (ORFs) from chimeric reads. (**A**) Heatmaps reflect the differentially expressed viral genes between two distinct strains. Non-canonical junctions provided the generation of variant open reading frames. (**B**) The stacked bar chart plots the proportion of reads in each cluster as estimated by variants. The truncated peptides derived from both in-frame or alternative frames of annotated ORFs expanded the coding capacity of PRRSV. (**C**) Overview of novel putative nested ORFs in viral genomes. The overlapping annotation data supporting predicted peptides are depicted using a Venn diagram.

Varied expression efficiency of individual ORF was common in distinct PRRSV isolates. To assess the coding capacity of the PRRSV transcriptome, the total amount of sg mRNAs produced by pooling multiple TRS-B encoding the same ORF was used to indicate the expression abundance of that ORF. Among the XM-2020, ORF7 (N, 70.82%) had the highest abundance, followed by ORF6 (M, 8.84%) and ORF5 (GP5, 8.17%). Whereas in GD, the most abundant was ORF7 (N, 48.30%), followed by ORF5 (M, 26.77%) and ORF6 (GP5, 16.85%) (Figure 8A). Alternatively, the 3′ splicing site of certain sg mRNAs was mapped to the C-terminal region of the annotated gene, which may result in the translation of internal ORF or downstream ORF. Figure 8B reveals the distribution of these theoretical variants and which type they are derived from. Overall, 26 and 17 putative peptides were identified in the XM-2020 and GD transcriptomes, respectively (Figure 8C). Of note, the emerging evidence indicated that a novel alternative open reading frame was discovered in XM-2020, with lower transcriptional levels, encoding a 37-amino acid peptide inside the coding region of the N gene, named ORF7a (Table S3). This finding was supported by another study, and further experimental strategies are needed to validate the functional relevance of this potential short peptide during PRRSV infection [32,33]. Although the authentic expression profiles of these N-terminal truncated ORFs and their potential functions remain unclear, they represent an additional level of transcriptome complexity and greatly expand our earlier knowledge of the coding capacity for PRRSV. It is also worth noting that some non-full-length peptides may be assembled into viral particles and continue to spread, so it is necessary to keep vigilance to detect genomic rearrangements and deletions.

### 3.6. Revealing m5C Sites in gRNA and sg mRNAs

**Figure 9:**
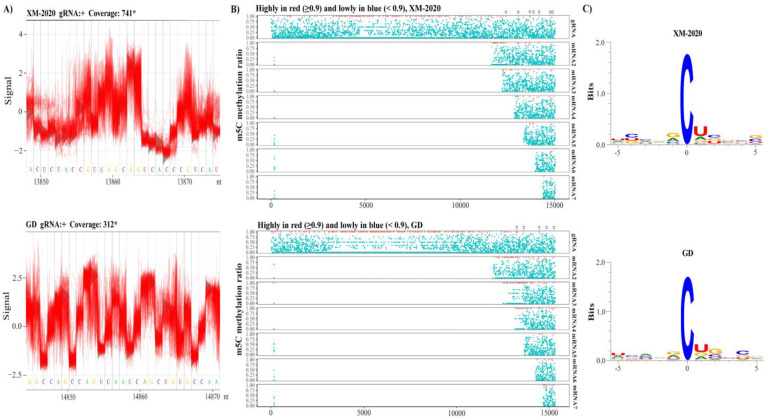
Potential profiles of 5-methylcytosine RNA modifications in PRRSV. (**A**) Close-up view of the raw electrical signals from nanopore sequencing. (**B**) Characterization of m5C modification in the transcriptome range. The red dot highlights the predicted sites with a methylation score > 0.9. The asterisk represents the consistent methylation modifications between genomic RNA and the nested sg mRNAs. (**C**) Motif analysis of 5 nt upstream and downstream of m5C sites across the PRRSV genome.

Methylated cytosine (m5C) has been identified as a widespread epigenetic marker in various RNAs, and such modifications have recently been deemed as pivotal regulators of gene expression at the post-transcriptional level, playing key roles in regulating processing, stability, and splicing of mRNAs. Through the detection of deviations in voltage signals, direct RNA-seq can reveal the complexity of modification of mRNA (Figure 9A). This study depicted the first comprehensive atlas of cytosine methylation in the epitranscriptome during the early stages of PRRSV infection in vitro. Both viruses showed the widespread occurrence of m5C in poly(A) RNA. The data revealed substantial differences in the number and distribution pattern of m5C in different viruses, with the nsp2 regions exhibiting high modification site density in both XM-2020 and GD (Figure 9B). Based on Tombo, 43 and 31 high-confidence m5C sites were identified in XM-2020 and GD, respectively (frequency > 0.9, covered > 10 reads); nevertheless, no obvious motif could be detected around ‘C’ (Figure 9C). Furthermore, we sought to determine whether there is a potential m5C location preferentially existing in sg mRNA clusters. A pronounced increase of the m5C frequency near the initial of the 3’ splice sequences was observed in both viruses, suggesting this modification was preferentially enriched around the translational start codon of sg mRNAs. Notably, multiple 5-methyl cytosine modification sites consistent with the genomic results were seen in sg mRNA clusters (Figure 9B). These results propose that the highly consistent methylation modifications may play the same functional role in different transcript classes. Overall, this work demonstrated the potential association of methylation modifications and transcription regulation and provided a framework for future research toward the role of epigenetics in the evolution and pathogenicity of PRRSV. 

## 4. Discussion

Efficient and sophisticated transcription strategies are fundamental for PRRSV to replicate, assemble, and quickly respond to the host cell environment. Accordingly, decoding the transcriptional atlas and understanding their regulation mechanisms is vital to elucidating the key biological strategies of disease control. Traditionally, most transcriptomics studies of PRRSV still rely on identifying sg mRNAs through Northern blot methods or Illumina sequencing [34,35,36]. Despite the growing interest in the potential of transcriptome analysis, few or no studies have directly addressed recombination events or TRS-B systematic annotation in PRRSV. This could be related to the limitations of conventional sequencing technologies, such as the inability to identify full-length splice isoforms that span regions. Currently, Nanopore direct RNA sequencing (ONT) offers a single ultralong read length that can facilitate the reconstruction of complex genomes and analysis of rare transcriptional patterns [20]. This platform has been extensively used in transcriptomics research targeting SARS-CoV-2 and captured a significant number of spliced mRNAs with high resolution [37,38,39]. Nonetheless, Nanopore reads usually have a relatively high error rate, limiting their reliability and usefulness. Overcoming these obstacles will require further breakthroughs in developing new nanopores, increasing throughput, optimizing base-calling methods, and bioinformatics pipelines [40]. Once these constraints are fixed, ONT will have a tremendous impact on the field of genomics. 

By using state-of-the-art nanopore sequencing, this dataset offered the most systematic and comprehensive landscape of the PRRSV transcriptome yet achieved. For all viruses tested, these splicing events predominantly take place between TRS-B upstream of individual ORF and TRS-L. However, a small fraction of RNA fusions might be produced with a TRS-independent pattern. Intuitively, TRS-independent populations from GD were more diverse than those from XM-2020, indicating a narrow selectivity in the packaging process. Additionally, we found that aberrant non-canonical populations were generally heterogeneous and tended to be enriched around canonical breakpoints, which was presumably caused by the instability of the RNA-dependent RNA polymerase “jump” between homologous sequences. Several early studies described PRRSV non-canonical sg mRNAs (termed heteroclite subgenomic RNAs), with their packaging signals more biased to splice in various sites within ORF1a, forming a diffuse pattern of fusions [41,42,43]. The subsequent experiments indicated that atypical RNAs were intrinsically associated with PRRSV infection and were co-packaged into infectious particles with gRNA [44]. Heteroclite RNAs may also be involved in the establishment and maintenance of viral persistence infections, although the mechanism remains to be determined [45,46,47]. It is noteworthy that a similar observation has been reported for SARS-CoV-2, implying that the production of TRS-independent transcripts was seen as a common programmed phenomenon involving potentially biological functions rather than species specificity [37]. Incomplete viral RNAs were also considered as defective interfering particles (DVG) during natural infections, thereby limiting the replication of standard viruses [48,49]. Recent work has begun to elucidate the distinct functions of these different DVGs, such as promoting the persistence and maintaining the immunostimulatory activity of a wild-type virus infection [50]. Further study is required to determine the origin of the new heterogeneous subpopulation and the exact mechanisms behind recombination events. 

TRS-B is critical for controlling transcription cycle gene expression; thus, its identification and analysis is the first step toward understanding its transcriptional relevance. Multiple distinctly spliced sg mRNA transcripts coding for the same protein have been documented, with the TRS with the highest utilization (i.e., the highest abundance) being referred to as the major, while others with low-level usage were referred to as alternative TRSs [17,18]. XM-2020 showed more alternative TRS sites during infection. These observations strongly suggest that the alternative TRS’s variation among virus subtypes was adaptive to maximize its efficiency of transcription under stress conditions. We cannot say if the increased utilization of TRS-B sites is the cause or consequence of more efficacious virus assembly, but our findings are essential to explore the potential adaptations correlated with viral evolutionary or persistent infection. Studies on transcriptomic data also uncovered additional TRS-B that originate from internal regions of known mRNA-encoding genes, which may result in the expression of N-terminally truncated polypeptides. It has been reported that the translation of particular downstream ORFs might be related to enhancing the translation of the canonical ORF, and the increasing number correlated positively with their regulatory effect [51,52]. In this sense, this expansion added extensive complexity to transcriptional regulation and its coding potential of PRRSV.

Doubtlessly, the field of epitranscriptomics was uncovered around dynamic RNA modifications and their roles in viral transcription at a rapid pace [53]. Currently, advances in Nanopore technology have enabled unbiased detection of RNA modification and its dynamics. Here, we first provided a single-nucleotide resolution mapping of cytosine methylation in poly(A) RNA with these two different strains, and further explored the high correlation of modification sites between sg mRNAs and genomic RNA. RNA methylation differed extensively between strains of the same geographical origin, whereas the molecular mechanisms that underlie genetically driven epigenetic heterogeneity are still largely unknown. This conservative pattern of RNA methylation is indicative of a heritable non-random process, and thus can serve as a repressive or activation marker to maintain gene expression in homeostatic balance. Dynamic RNA modifications have emerged as a crucial, but widely neglected, part of transcriptional regulation in PRRSV. Collectively, the database provided a valuable resource for future studies regarding the functional impact of m5C on genomics and transcriptomics.

## 5. Conclusions

Longitudinal monitoring of sg mRNA expression within the host and the profiles of TRS-B sites can provide valuable information for exploring the molecular mechanism of genetic evolution selection and quasispecies diversity in PRRSV. Here, we combined Nanopore long-read technology and next-generation short-read sequencing methods to investigate genome-wide full-length splice isoforms and accurately reconstruct the internal transcriptome architecture. This hybrid solution can make up for the weaknesses of their respective technologies, allowing for more accurate elucidation of the transcriptome complexity. As the third-generation sequencing appears, revealing the transcriptional regulation mechanism of the PRRSV will help researchers to track and eventually curb it. 

## Data Availability

The raw sequencing data can be downloaded from the NCBI sequence read archive (SRA) under the accession number: SRR16250047, SRR16250046, SRR16190334, and SRR16190333. The data that support the findings of this study are available from the corresponding author upon reasonable request.

## Supplementary Materials

The following are available online at https://www.mdpi.com/article/10.3390/v13122531/s1, Figure S1: PRRSV replication in PAMs, Figure S2: Distribution of differential subset of transcripts, Figure S3: Correlation between TRS duplex stability and template switching, Table S1: PRRSV different strains used in this study, Table S2: For each spanning-junction read, 5′ and 3′ selections were recorded in the Nanopore Data, Table S3: This table extends the information for the recombination events dependent on TRS-L in XM-2020 DRS data, Table S4: This table extends the information for the recombination events dependent on TRS-L in GD DRS data.
