# Supplementary figures and images for: Nanopore-Based Direct RNA-Sequencing Reveals a High-Resolution Transcriptional Landscape of Porcine Reproductive and Respiratory Syndrome Virus
Source: Viruses. 2021 Dec 16;13(12):2531. doi: 10.3390/v13122531 (PMC8706258; doi:10.3390/v13122531)

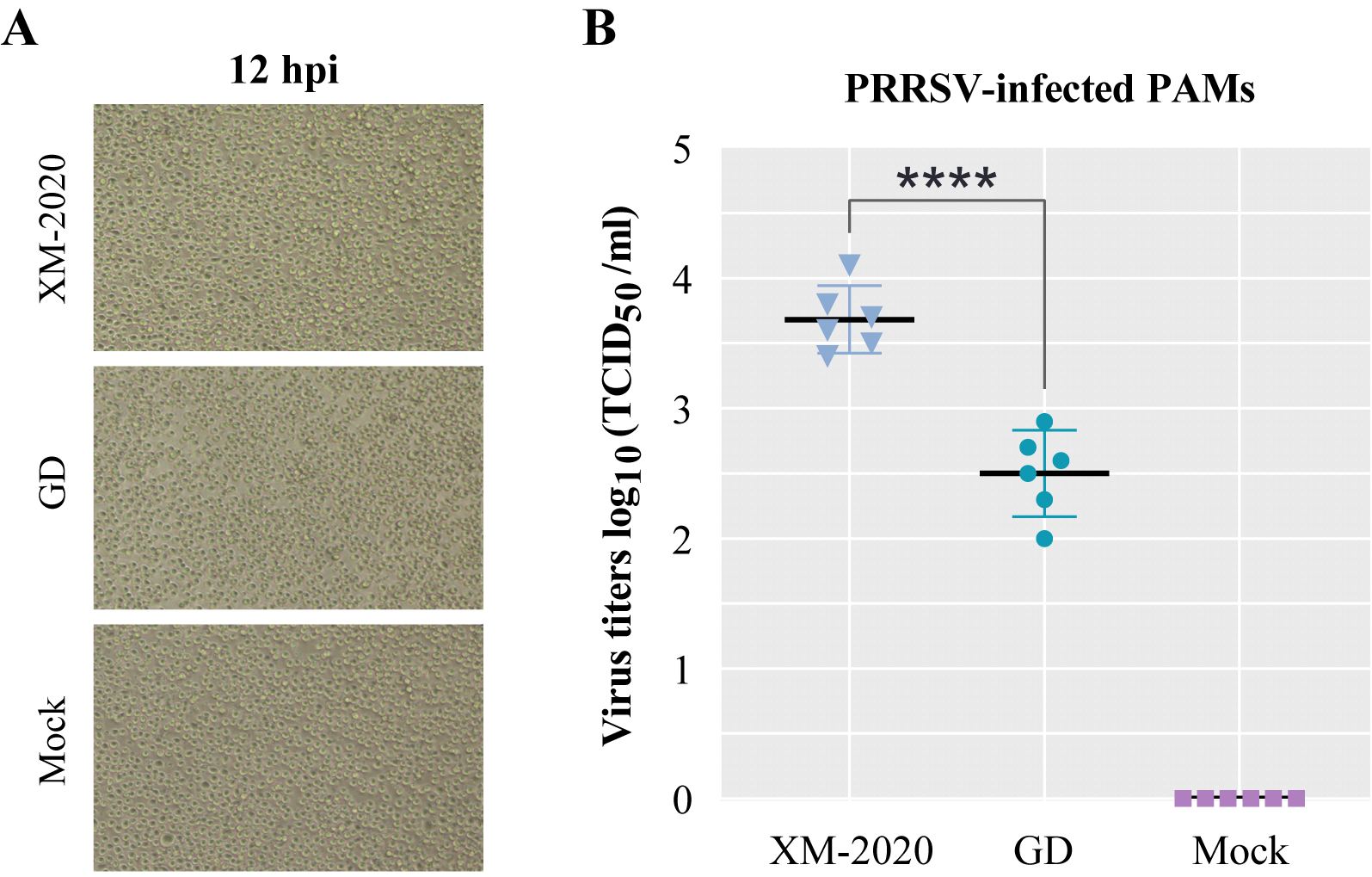

Supplement: Supplementary file 1 [file viruses-13-02531-s001.zip › viruses-1438771-supplementary/Supplementary File/Figure/Figure S1.tif]

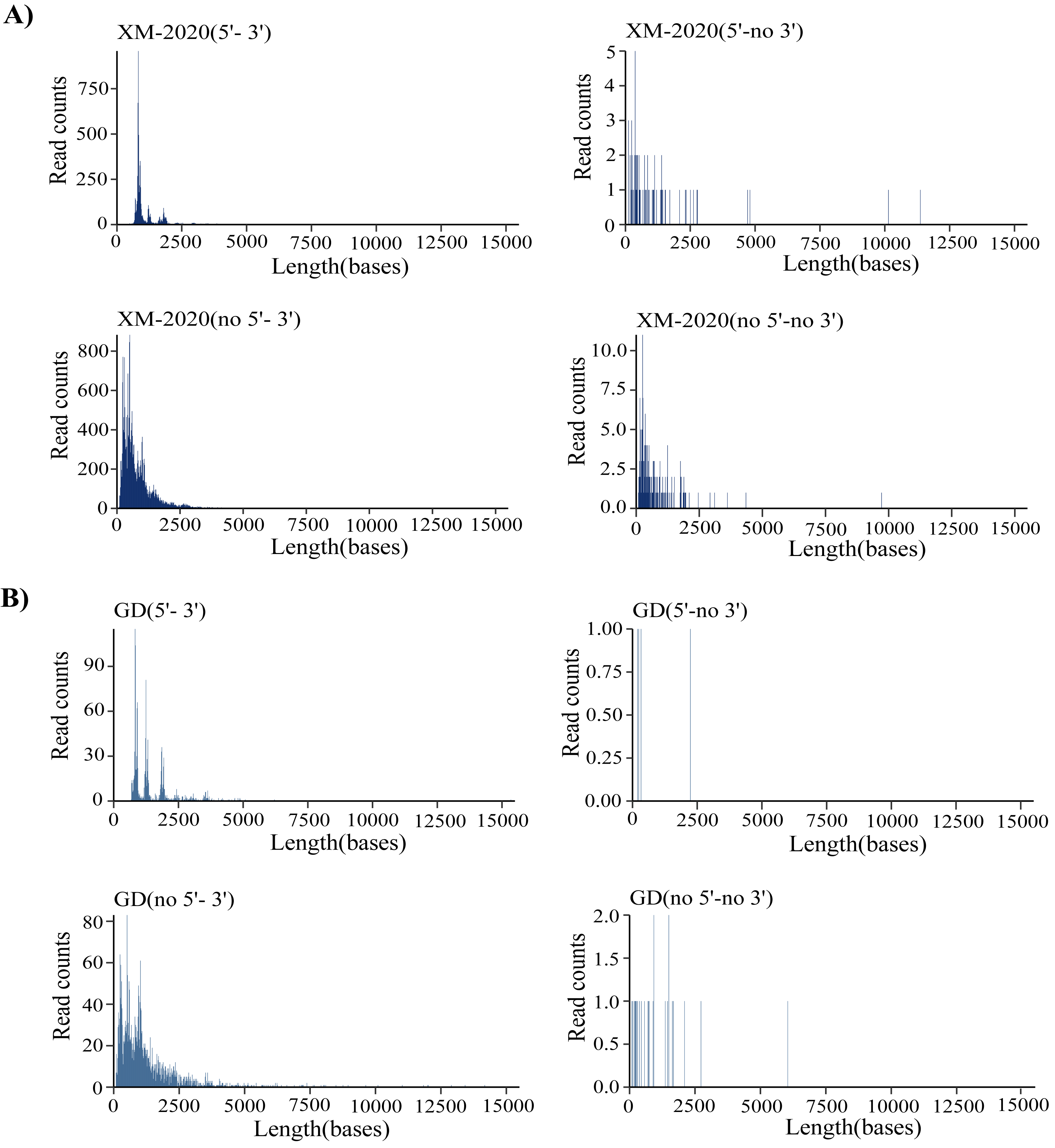

Supplement: Supplementary file 1 [file viruses-13-02531-s001.zip › viruses-1438771-supplementary/Supplementary File/Figure/Figure S2.tif]
